# Rapid adaptation drives invasion of airway donor microbiota by *Pseudomonas* after lung transplantation

**DOI:** 10.1038/srep40309

**Published:** 2017-01-17

**Authors:** M. Beaume, T. Köhler, G. Greub, O. Manuel, J-D. Aubert, L. Baerlocher, L. Farinelli, A. Buckling, C. van Delden, The Swiss Transplant Cohort Study, Rita Achermann, Patrizia Amico, Philippe Baumann, Guido Beldi, Christian Benden, Christoph Berger, Isabelle Binet, Pierre-Yves Bochud, Elsa Boely, Heiner Bucher, Leo Bühler, Thierry Carell, Emmanuelle Catana, Yves Chalandon, Sabina de Geest, Olivier de Rougemont, Michael Dickenmann, Michel Duchosal, Thomas Fehr, Sylvie Ferrari-Lacraz, Christian Garzoni, Paola Gasche Soccal, Emiliano Giostra, Déla Golshayan, Daniel Good, Karine Hadaya, Jörg Halter, Dominik Heim, Christoph Hess, Sven Hillinger, Hans H. Hirsch, Günther Hofbauer, Uyen Huynh-Do, Franz Immer, Richard Klaghofer, Michael Koller, Bettina Laesser, Roger Lehmann, Christian Lovis, Hans-Peter Marti, Pierre Yves Martin, Luca Martinolli, Pascal Meylan, Paul Mohacsi, Isabelle Morard, Philippe Morel, Ulrike Mueller, Nicolas J Mueller, Helen Mueller-McKenna, Antonia Müller, Thomas Müller, Beat Müllhaupt, David Nadal, Manuel Pascual, Jakob Passweg, Chantal Piot Ziegler, Juliane Rick, Eddy Roosnek, Anne Rosselet, Silvia Rothlin, Frank Ruschitzka, Urs Schanz, Stefan Schaub, Christian Seiler, Susanne Stampf, Jürg Steiger, Guido Stirnimann, Christian Toso, Dimitri Tsinalis, Jean-Pierre Venetz, Jean Villard, Madeleine Wick, Markus Wilhelm, Patrick Yerly

**Affiliations:** 1 Service of Infectious Diseases, University Hospitals of Geneva and Department of Microbiology and Molecular Medicine, University of Geneva, Geneva, Switzerland; 2 Institute of Microbiology, Lausanne University Hospital and University of Lausanne, Lausanne, Switzerland; 3 Service of Infectious Diseases, Lausanne University Hospital and University of Lausanne, Lausanne, Switzerland; 4 Transplantation Center, Lausanne University Hospital and University of Lausanne, Lausanne, Switzerland; 5 Service of Pulmonary Diseases, Lausanne University Hospital, Lausanne, Switzerland; 6 Fasteris SA, Plan-les-Ouates, Switzerland; 7 ESI & CEC, Biosciences, University of Exeter, Penryn Campus, Cornwall, United Kingdom; 8 Basel University Hospitals, Basel, Switzerland; 9 Geneva University Hospitals, Geneva, Switzerland; 10 Bern University Hospitals, Bern, Switzerland; 11 Zurich University Hospitals, Zurich, Switzerland; 12 Kantonsspital, St Gallen, Switzerland; 13 Lausanne University Hospitals, Lausanne, Switzerland; 14 University of Lausanne, Lausanne, Switzerland

## Abstract

In cystic fibrosis (CF) patients, chronic airway infection by *Pseudomonas* leads to progressive lung destruction ultimately requiring lung transplantation (LT). Following LT, CF-adapted *Pseudomonas* strains, potentially originating from the sinuses, may seed the allograft leading to infections and reduced allograft survival. We investigated whether CF-adapted *Pseudomonas* populations invade the donor microbiota and adapt to the non-CF allograft. We collected sequential *Pseudomonas* isolates and airway samples from a CF-lung transplant recipient during two years, and followed the dynamics of the microbiota and *Pseudomonas* populations. We show that *Pseudomonas* invaded the host microbiota within three days post-LT, in association with a reduction in richness and diversity. A dominant mucoid and hypermutator *mutL* lineage was replaced after 11 days by non-mucoid strains. Despite antibiotic therapy, *Pseudomonas* dominated the allograft microbiota until day 95. We observed positive selection of pre-LT variants and the appearance of novel mutations. Phenotypic adaptation resulted in increased biofilm formation and swimming motility capacities. *Pseudomonas* was replaced after 95 days by a microbiota dominated by *Actinobacillus*. In conclusion, mucoid *Pseudomonas* adapted to the CF-lung remained able to invade the allograft. Selection of both pre-existing non-mucoid subpopulations and of novel phenotypic traits suggests rapid adaptation of *Pseudomonas* to the non-CF allograft.

Lung transplantation (LT) is recognized as the ultimate therapeutic intervention for cystic fibrosis (CF) patients with end-stage lung disease. Unfortunately, allograft infections and development of chronic rejection lead to loss of graft function in about 50% of patients at five years^1^. CF lung-recipients suffer from chronic pre-transplant infections with microorganisms including *Staphylococcus aureus, Burkholderia cepacia,* and *Pseudomonas aeruginosa,* the latter predominating in adults^2^. *P. aeruginosa* outcompetes other bacteria and remarkably adapts to the particular CF-airway microenvironment by accumulating mutations over years^3^. Whereas lung transplantation removes the major infectious site, *Pseudomonas* potentially residing in the recipient’s sinuses may seed the allograft^4^,^5^. The success of *Pseudomonas* in colonizing the allograft airways depends on antibiotic treatments, the host response hampered by immunosuppressive therapies and initial mucosal damage^6^, as well as inter-species competition. Despite this challenging situation, allograft airway colonization by *Pseudomonas* is frequent^7^.

Little is known to date about the microbiota of lung transplant recipients. It remains unclear whether particular pre-LT infections/colonization are associated with poor post-LT outcomes. Several studies demonstrated that CF-patients infected by *P. aeruginosa* had more post-LT respiratory infections than non-infected non-CF patients^8^,^9^. However, no significant difference in survival was reported. In addition, CF-patients infected with pan-resistant microorganisms seem to have a reduced post-LT survival compared to patients infected with antibiotic-susceptible bacteria^10^. Other studies using culture-independent methods suggested differences between the microbiota of CF as compared to non-CF lung transplant recipients^11^ and healthy control patients^12^, mainly due to the ubiquity and dominance of *Pseudomonas* in CF-patients^11^.

To explain the successful allograft colonization by *Pseudomonas* we can envisage that the CF-adapted strains also survive in a non-CF allograft environment, or that *Pseudomonas* can adapt rapidly to the novel physiological and microbiological conditions prevailing in the allograft. Here, we attempt to distinguish between these hypotheses by following the population dynamics, and by defining the real-time phenotypic and molecular evolution of *Pseudomonas* post-transplant. Specifically, we investigated longitudinal airway samples from both intermediate (conductive zone) and distal (respiratory zone) sites in a CF-lung transplant recipient chronically infected by *P. aeruginosa* before transplantation and transiently thereafter, while simultaneously characterizing changes in the co-colonizing microbiota. We find compelling evidence for major changes in the allograft microbiota and for rapid adaptation of *Pseudomonas* population to the allograft.

## Results

### Pseudomonas colonized the conductive allograft airways in the first days after LT

**Figure 1:**
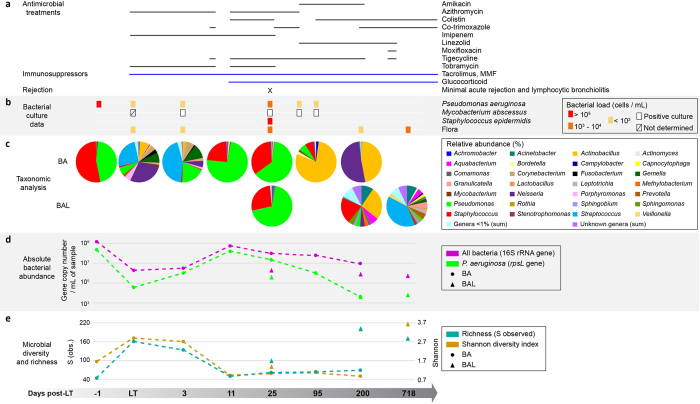
Evolution of the microbial airway composition after LT. Shown are the sequential analysis of bronchial aspirates (BA) and bronchoalveolar lavages (BAL) collected pre- and post-LT. (**a**) Timing of antimicrobial and immunosuppressing therapies. (**b**) Bacterial cultures over time. Bacterial load (number of cells/mL) is represented as following: red square, >10^5^; orange square, 10^3^–10^4^; yellow square, <10^3^; white square, positive culture; crossed square, not determined. (**c**) Taxonomic analysis, cutoff set >1%. (**d**) Absolute bacterial abundance determined by performing qPCR on gDNA extracted from native airway samples. qPCR detection limit established at 10^3^ *rspL* copy number/mL of sample. (**e**) Microbial diversity and richness.

We studied the dynamics of total microbial communities of the allograft conductive and respiratory airways using 16 S rRNA gene sequencing of one pre-LT and six successive post-LT bronchial aspirates (0, 3, 11, 25, 95, 200 days), as well as of three post-LT bronchoalveolar lavages (25, 200 and 718 days)(Table 1). Bacterial community diversity and richness were determined by the Shannon-Weaver and the S indexes respectively^13^. *Pseudomonas* and *Staphylococcus* genera dominated in the bronchial aspirate (BA) before LT (day −1) with elevated bacterial counts, and low Shannon and S indexes (Fig. 1c,d and e). The BA at the time of LT (day 0) reflected the donor airway microbial flora with a completely different distribution (Fig. 1c). Both indexes were elevated indicating a high microbial diversity and richness, typically characterizing healthy lungs^12^ (Fig. 1e). This sample already contained *P. aeruginosa*, detectable both by qPCR and bacterial culture. At D3 post-LT, abundance of the *Pseudomonas* genus had already increased in a BA (Fig. 1c and d) and the pre-LT distribution was restored 11 days after LT, with *de novo* colonization of the graft airways by *Pseudomonas* and *Staphylococcus* genera (Fig. 1c). This shift went along with an increase of total bacterial and *P. aeruginosa* loads, despite targeted antibiotherapy (Fig. 1d). Shannon and S values of the BA dropped close to pre-LT levels, indicating a decrease of bacterial diversity and richness in the conductive airways of the transplant (Fig. 1e). The predominance of these two pathogens was maintained for one month after LT (day 25) in both BA and BAL, although *Pseudomonas* and total bacterial abundance decreased 10 fold. Bacterial culture data were consistent with taxonomic analyses of the microbial communities, showing a high abundance of *Pseudomonas* between day three and 25 post-LT (Fig. 1b). At day 95, the situation had inversed and several members of the commensal flora such as *Actinobacillus* and *Bordetella* had efficiently outgrown *Pseudomonas* and *Staphylococcus* in the BA (Fig. 1c,d). After six months (day 200), *Pseudomonas* represented less than 0.17% of all bacterial taxons, a result confirmed by qPCR (Fig. 1d). At this point, the BA was mainly colonized by *Actinobacillus* and *Neisseria* genera, with low Shannon and S values. This contrasted with a high microbial diversity and richness in a concomitant BAL (Fig. 1e). Despite aggressive antibiotic treatments the absolute abundance of total bacteria did not vary drastically after transplantation, showing only a 10-fold decline between day 25 and 200. Note that colonization by *Mycobacterium* disappeared after day 95 in BA and day 200 in BAL (Table S1).

### Population dynamics and evolution of Pseudomonas isolates after LT

**Figure 2:**
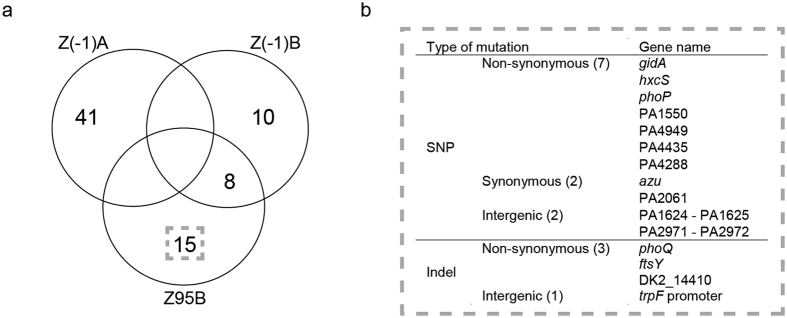
Genome comparison between two pre-LT and one late post-LT isolate. (**a**) Venn diagram summarizing the number of mutations identified in each genome. (**b**) Description of the 15 mutations detected in the genome of the final post-LT isolate Z95B.

**Figure 3:**
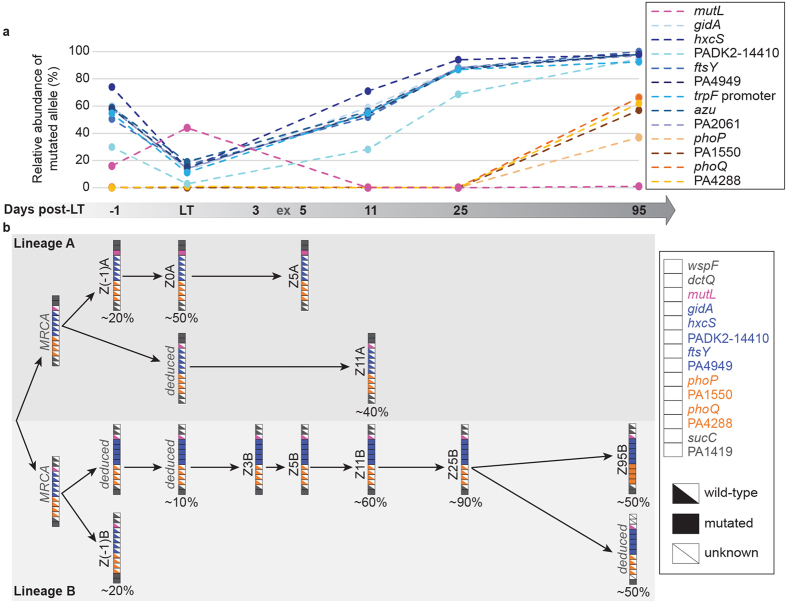
*In patient* evolution of *Pseudomonas* genome variants after LT. (**a**) Relative abundance of mutated variants in the whole *Pseudomonas* population. Thirteen genes were amplified by PCR and amplicons were submitted to deep sequencing. Mutations previously identified in the Z95B genome were searched in the total pool of sequencing reads. The amount of wild-type and mutated versions of the gene are expressed as the percentage of mutated alleles in the total population for each gene. (**b**) Evolutionary history of *Pseudomonas* isolates and populations following LT. The vertical arrays show the status (wild-type or mutated) of each gene in strains isolated from bronchial aspirates. These data analyzed concomitantly to those obtained for the total population (panel A) allows to reconstruct the evolution of the isolates, and to determine their respective percentage of variants in the total *Pseudomonas* community. The status of the hypothetical intermediate genomes (*deduced*) and those of the most recent common ancestors (*MRCA*) were inferred from all data taken together. ex = extubation.

To determine if pre-LT and post-LT isolates had the same genotypes, we performed both Random Amplification of Polymorphic DNA (RAPD) and a more discriminative analysis using the Clondiag array^14^. Genotypic analysis confirmed that the same unique genotype was recovered before and after LT from the conductive airways (Fig. S1). We investigated whether the passage from the CF-environment (sinuses or other recipient body sites) to the non-CF-environment (airways of the donor allograft) resulted in rapid adaptive evolution by first sequencing the genomes of two pre-LT *Pseudomonas* isolates (Z(−1)A and Z(−1)B), as well as the last available post-LT isolate at day 95 (Z95B). A total of 74 mutations were identified by comparing the genomes of these three isogenic strains (Fig. 2a, Tables S2 and S3). The strain isolated at day 95 showed 15 mutations (11 SNPs, three deletions and one insertion) that were absent in both pre-LT isolates (Fig. 2a). Ten of these were non-synonymous, and three were in intergenic regions, suggesting positive selection acted on at least some of these mutations, although genetic hitchhiking may explain the increase in frequency of others (Fig. 2b). The mutations occurred in genes coding for proteins belonging to several functional categories: regulators (37%), cell metabolism (18%), cell cycle control (18%), hypothetical proteins (18%), transport and secretion (9%). Z95B shared eight mutations with Z(−1)B, but none with Z(−1)A. We further quantified by deep sequencing the relative amount of 13 alleles mutated in strain Z95B within the native bronchial aspirates. This allowed us to follow both the emergence and the evolution of each mutation within the whole *Pseudomonas* population over a 3 months period (Fig. 3). We identified two distinct sub-populations. The first one, already present pre-LT, decreased from 60 to 20% at the time of transplantation, and then rapidly increased after day three in the allograft airways to reach 90–100% of the total *P. aeruginosa* population at day 95 (blue lines in Fig. 3a). The second sub-population, absent before and at the time of LT, remained undetectable until day 25 post-LT and reached about 40 to 60% at day 95 (brown-orange lines in Fig. 3a). These differences in frequency through time were all significant with a sequencing coverage comprised between 5’541 and 513’882 reads per sample per target. Of notice the Z(−1)A genome contained a non-synonymous SNP in the *mutL* gene, encoding a DNA mismatch repair protein. Mutations in *mutL* have been associated with the hypermutator phenotype showing a high prevalence in chronic respiratory infections^15^. This variant of the *mutL* gene initially increased and then disappeared at day 11 post-LT (Fig. 3a).

**Figure 4:**
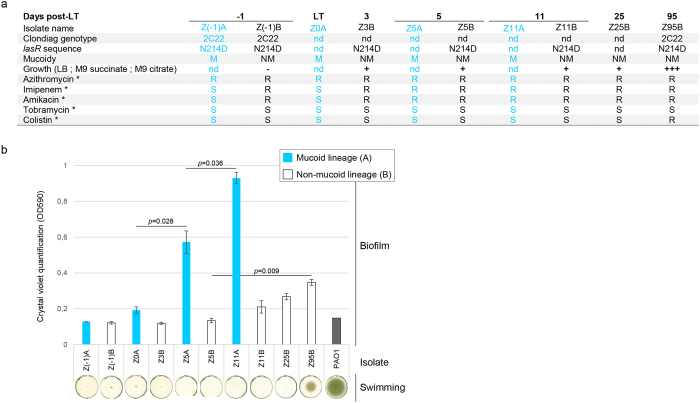
Phenotypic analysis of ten *P. aeruginosa* strains isolated from sequential airway samples. (**a**) *indicate the antibiotic resistance (R) or susceptibility (S) phenotypes according to EUCAST breakpoints (minimal inhibitory concentrations are provided in Table S4). (**b)** Biofilms were stained with crystal violet after 24 hours of static growth. Optical densities were measured at 590 nm. Biofilm formation of the reference strain PAO1 is shown for comparison. Error bars showed standard deviations; statistical significance was calculated by an unpaired t-test. Swimming phenotype was evaluated after 22 h of growth at 37 °C in LB 0.2% agar.

We further explored the post-LT evolutionary dynamics of mutations by characterizing specific genotypes and their phenotypes. We performed capillary sequencing on PCR products amplified from gDNA of ten isolates collected between the day before transplantation (D−1) and D95 post-LT. We focused on non-synonymous mutations absent in the two ancestor isolates Z(−1)A and B, but present in Z95B (*gidA, hxcS, ftsY*, PADK2–14410, PA4949, *phoP, phoQ*, PA1550, PA4288), as well as on mutations present in only one of the two ancestor genomes (*mutL, dctQ, wspF, sucC*, PA1419) (Fig. 3b). We were able to identify two independent lineages. Lineage A was characterized by a mucoid phenotype, probably due to a mutation in the *algU* promoter, and by mutations in *wspF* and *dctQ*. Isolates Z(−1)A, Z0, Z5A and Z11A seemed all to be descendants of a common ancestor carrying the *wspF* and *dctQ* mutations. Isolate Z11A probably derived directly from this ancestor, whereas isolate Z(−1)A accumulated an additional mutation in *mutL* leading to a second sub-lineage including Z0 and Z5A. Lineage A reached about 40% of the total *Pseudomonas* population at day 11, but then disappeared at day 25. Lineage B was characterized by a non-mucoid phenotype and a mutation in ORF PA1419. This lineage represented 10% of the population at the time of transplantation, but was dominant after day 11 (Fig. 3b). It further split into two sub-lineages: the first one, represented by isolate Z(−1)B was not retrieved at a later time point. It carried a mutation in *sucC*, and was unable to grow on succinate (Fig. 4a), a main carbon source of *Pseudomonas*. The second sub-lineage, harboring mutations in *gidA, hxcS, ftsY*, PADK2-14410 and PA4288, increased in proportion and represented 90% of the population at day 25. Three months post-LT, a new variant characterized by additional mutations in *phoP, phoQ*, PA1550 and PA4288 emerged from this sub-lineage. Deep sequencing data demonstrated that two subpopulations, derived from lineage B, co-existed with similar proportions three months after LT, suggesting a positive selection for *gidA, hxcS, ftsY*, PADK2-14410, and PA4949 mutations.

Phenotypic analysis of post-LT isolates showed a progressive increase in biofilm formation capacity both in mucoid and non-mucoid lineages (Fig. 4b). In contrast, we observed the appearance of swimming motility^16^ only in the last isolate (Z95B) of the non-mucoid lineage B (D95, Fig. 4b). This isolate also showed increased growth capacity in LB, M9 succinate and M9 citrate media (Fig. 4a). All isolates were susceptible to tobramycin. Imipenem and amikacin resistant isolates appeared in the mucoid lineage A at day 5, whereas non-mucoid isolates were already resistant to these antibiotics before LT. Despite early colistin treatment, we observed resistance to colistin only at day 95 in the non-mucoid lineage B (Fig. 4a and Table S4).

## Discussion

It has previously been unclear whether: i) colonization of the lung allograft by CF-adapted populations of *Pseudomonas* occurs because the allograft does not represent a particularly novel environment hence the populations are already well adapted, or ii) the allograft represents a new environment (both in terms of recipient physiological and immunological status, and donor lung physiology and microbiota) to which *Pseudomonas* must adapt. We observed that, despite aggressive antimicrobial therapy and the preexisting donor microbial flora, *Pseudomonas* rapidly colonized the allograft after lung transplantation, in association with a drastic reduction of both microbial richness and diversity. Colonization was associated with rapid adaptive evolution of *Pseudomonas*, resulting from selection of a pre-existing sub-population and *de novo* mutations that emerged later during allograft colonization. Although we cannot exclude that some mutations hitchhiked along with those under selection, the very low proportion of synonymous mutations and the large population size rules out genetic drift as a major driver of evolution.

What were the likely phenotypic consequences of the selected mutations, and why did they confer fitness benefits or costs? We speculate that the *sucC* mutation was not detected post-LT, since it potentially prevented growth on succinate. Mucoid isolates and hypermutators (*mutL*-mutants) were both highly prevalent in the CF-lung environment^15^,^17^, and these isolates were probably the first to seed into the allograft from the CF-host environment. However, both phenotypes disappeared after day 11. We speculate that these CF-associated phenotypes were not required for persistence of *Pseudomonas* in its new environment. It has been hypothesised that the mucoid phenotype could protect *Pseudomonas* against host inflammatory defense mechanisms^18^. Maintenance of a mucoid phenotype might therefore not be favored by the post-transplant immunosuppressive therapy. In CF patients, *P. aeruginosa* isolates commonly acquire mutations in AlgU and MucA leading to overproduction of alginate (mucoid phenotype), associated with a decline in lung function^19^. Several studies showed that alginate elicits opsonic antibody production and thus has strong immunogenic properties^20^,^21^. Mucoid isolates might have been counter-selected because of their high immunogenicity or because of environmental conditions^22^. Interestingly, the *Pseudomonas* biomass decreased along with the disappearance of the mucoid lineage.

Seven of the mutations were present at relatively high frequencies prior to LT but increased to almost 100% after three months. Mutations in *gidA* were shown to have important phenotypic effects: deletion of *gidA* reduced the virulence of several Gram-negative bacteria^23^, and was associated with down-regulation of *rhl quorum-*sensing in *P. aeruginosa*^24^. The advantage of some of the mutations that aroused *de novo* in the allograft environment is more obvious: *phoP* and *phoQ* mutations could be associated with colistin-resistance^25^,^26^, as well as reduced cytotoxicity^27^. Indeed, deletion of *phoQ* is one the most frequent mutational events linked to colistin resistance^28^. Interestingly previous work showed significantly impaired competitiveness of *phoQ*-mutants in a chronic rat lung infection model^29^. Selection of these mutations after LT clearly suggests that adaptation can occur in the graft.

We also observed a progressive increase of biofilm formation in the two lineages. This phenotype seems therefore particularly important initially for successful allograft colonization. The biofilm mode of growth is involved in persistence and antibiotic resistance^30^. The fact that biofilm formation increased independently in two different lineages during colonization suggests that genetic drift is unlikely to be responsible for this phenotype. At 95 days, increased swimming motility appeared as a *de novo*-acquired phenotype. Swimming motility and biofilm formation often oppose one another; our results suggest that both are not mutually exclusive. It is striking that swimming motility appeared when the *Pseudomonas* biomass was significantly declining. One could postulate that it represents a bacterial survival strategy enabling bacteria to reach nutritionally more favorable niches, although hitchhiking with other traits may also be possible.

On the day of transplantation, the allograft microbiota was characterized by a high richness and diversity probably reflecting a healthy donor organ. However, within eleven days *Pseudomonas* and *Staphylococcus* invaded the graft and established a completely different microbiota similar to the pre-LT composition. This observation agrees with previous reports showing reduced richness and diversity following LT^11^,^12^. Interestingly, the microbial communities shifted again three months post-LT with a dominance of *Actinobacillus*. In parallel, *Pseudomonas* abundance declined, and diversity as well as richness increased in the lower airway samples. The difference between BA and BAL samples at day 200 could reflect the heterogeneity inside the lungs.

Based on our data, we can only speculate why the « healthy » microbiota finally replaced the predominance of the initially successful *Pseudomonas* predominating in both conductive and respiratory airways during the first months post-LT. Several factors might have influenced this outcome, but we suggest that *Actinobacillus* might have outcompeted *Pseudomonas* following the end of the imipenem therapy. Of importance, all *Pseudomonas* lineages were resistant to the various antibiotics used (including imipenem). One appealing hypothesis is that broad-spectrum antibiotherapy misbalanced the competition between the donor microbiota and *Pseudomonas* in favor of the latter. One other explanation is that systemic antibiotics were responsible for the major decrease of bacterial richness and diversity at day 11, without any involvement of *P. aeruginosa*.

It is possible that the observed changes in the *Pseudomonas* population occurred independently of lung transplantation, hence the population did not adapt to the allograft at all. However, we believe this to be doubtful. While recent work has shown rapid turnover of *P. aeruginosa* genotypes in CF patients over a matter of months, these changes are the result of fluctuations in haplotypes^31^,^32^ and are not directional changes as observed here. Also, as suggested by some recent work^33^, the genotypic and phenotypic evolution observed in the isolates harvested from the allograft could have occurred initially in another recipient compartment. However, this seems unlikely to us given that all other recipient compartments, including the sinuses, remain a CF-environment continuously favouring a mucoid phenotype. Therefore, the disappearance of a mucoid phenotype in the allograft after day 11 post-LT suggests adaptation to the non-CF allograft microenvironment. Potentially whole genome sequencing, paired to deep-sequencing of the entire *Pseudomonas* population used in the present study detected adaptation to the non-CF allograft missed in other studies by specific-gene capillary sequencing, which does not offer the same analysis depth.

To our knowledge this study is the first to describe in detail the dynamics of *P. aeruginosa* adaptation during allograft colonization, due to the difficulty to obtain longitudinal clinical samples from lung transplant recipients.

In conclusion, our study shows that despite aggressive antibiotic therapy, colonization of the allograft by *Pseudomonas* occurred already in the first days after LT. This was associated with rapid adaptive evolution of the population and major changes in the pre-existing allograft microbiota. However, these rapid initial invasion and adaptation did not allow long-term persistence of *Pseudomonas* post-LT in this patient after 95 days. Broad-spectrum antibiotics might have favoured *Pseudomonas,* which competed with the donor microflora. Although limited to a single patient our results clearly show that rapid adaptation to the novel environment may explain the successful colonisation of the lung allograft by *Pseudomonas.* Future studies, including larger numbers of patients, will be crucial to understand the subtle interplay between invading *Pseudomonas* and donor microbiota, to develop novel strategies preventing acute and chronic allograft infections.

## Methods

### Patient Characteristics

A 32 year-old CF female patient chronically infected by *P. aeruginosa* received a bilateral lung transplant for end-stage lung disease in 2011 (Table 1). As 95% of solid organ transplant recipients in Switzerland, she was included in the Swiss Transplant Cohort Study (STCS)^34^. Clinical data reported in the STCS database included culture-based microbiology assessments, antibiotic and immunosuppressive treatments, as well as the clinical status of the patient. We collected eight bronchial aspirates (BA) and three bronchoalveolar lavages (BAL) before, at the time of LT, and at various time points thereafter during two years (Table 1). The total clinical follow-up was 3.25 years. Due to pre-transplant colonization by *Mycobacterium abscessus* the patient was treated for a prolonged period with a combination of imipenem, linezolid, tigecycline and moxifloxacin (Fig. 1a).

### Collection of native airway samples, DNA extraction and isolation of Pseudomonas

Sequential bronchial aspirates (BA) and bronchoalveolar lavages (BAL) were collected during routine surveillance bronchoscopies. BA consisted of respiratory secretions aspirated below the bronchial suture, and represent heterogeneous sampling from both lungs at the level of the conductive airways. One BA sample was collected six hours post-transplantation and was labeled D0. BAL fluid was obtained by aspiration following instillation of >100 ml of NaCl 0.9% through the bronchoscope wedged in a subsegmental bronchus, thus representing a more homogenous sample from a distal respiratory zone. One mL aliquots of native BA and BAL samples were frozen immediately after sampling at −80 °C. *Pseudomonas* strains were isolated from BA samples by the routine diagnostic microbiology laboratory of the university hospital of Lausanne. Total genomic DNA (gDNA) was extracted from native samples after chemical and mechanical lysis, using the DNeasy kit (QIAGEN) according to the manufacturer’s instructions.

### Microbial community analysis

The V4-V6 regions of the 16 S rRNA gene were amplified by PCR (primer sequences in Table S5) and sequenced using an Illumina MiSeq platform (Fasteris SA, Switzerland). Filtered reads were mapped onto the GreenGenes database^35^ and were assigned to specific genus level lineages. Sample richness and diversity were calculated using the Shannon and S indexes respectively, with the VEGAN package with normalized data sets.

### Quantitative real-time PCR (qPCR)

qPCR was performed as previously described^36^. DNA was amplified using the SYBR Green Quantitect Kit (QIAGEN) with 0.6 μM of 16 S rRNA or *rpsL* primers (Table S5). Gene copy numbers per mL of sample were determined comparing the obtained cycle threshold (Ct) to a standard curve performed with gDNA of *P. aeruginosa* reference strain PA14.

### Genome analysis of clinical isolates

#### Genotyping

*P. aeruginosa* genotyping was performed by random amplification of polymorphic DNA (RAPD) using primer 208 as previously described^37^. Strains were further genotyped using the *P. aeruginosa* Clondiag kit^14^.

### PCR for capillary sequencing or digestions

PCR was performed on bacterial lysates. Presence of mutations was determined by sequencing or digestion of the purified PCR products (Table S6).

### Whole-genome and deep sequencing

Genomic DNA was prepared from *P. aeruginosa* isolates using the DNeasy Blood and Tissue kit (QIAGEN). Multiplex PCRs of genes carrying mutated alleles were performed on gDNA of native airway samples. The Illumina HiSeq 2500 platform (Fasteris SA, Switzerland) was used for all sequencing processes. Assembly, mapping, variant calling and allelic counting are described in the online supplement.

### Phenotypic assays

#### Microtiter dish biofilm

Bacteria were grown in M63 medium supplemented with 0.2% glucose, 2 mM MgSO4 and 0.05% Casamino acids in a 24-well polystyrene microtiter dish (Costar) at 30 °C under static conditions. Staining was performed as previously described^38^ and absorbance was measured at 590 nm.

#### Swimming motility

Bacteria were inoculated by toothpick into a 0.2% LB agar plate and incubated at 37 °C during 22 h. Experiments were performed in duplicate.

#### Growth measurements

Cultures and pellets were prepared as described for the biofilm assay. LB medium or M9 medium containing 2 mM MgSO4 and supplemented with 20 mM succinate or 25 mM citrate were inoculated with bacterial suspensions (final OD_600_=0.2). Experiments were performed in duplicate.

#### Minimal inhibitory concentrations (MIC)

MIC were determined as previously described^39^.

### Nucleotide sequence accession

16 S rRNA gene sequence files were deposited in EMBL-ENA under the accession number PRJEB13210, whole genome sequence files under the number PRJEB14598 and deep sequencing on the *P. aeruginosa* population under the number PRJEB14611 (Supplementary Table S7).

Additional details on the material and methods are provided in the supplementary information.

### Ethical statement

Authorization to use all clinical samples for research purposes were obtained from the local ethical committee of the University Hospitals of Geneva (authorization CER 07–301), and the patient provided her written informed consent. All methods and experimental protocols were carried out in accordance with the approved guidelines.

## Additional Information

**How to cite this article**: Beaume, M. *et al*. Rapid adaptation drives invasion of airway donor microbiota by Pseudomonas after lung transplantation. *Sci. Rep.* **7**, 40309; doi: 10.1038/srep40309 (2017).

**Publisher's note:** Springer Nature remains neutral with regard to jurisdictional claims in published maps and institutional affiliations.

## Supplementary Material

Supplementary Data

## Figures and Tables

**Table 1:** Summary of CF-subject characteristics and samples.

Gender	Female
*CFTR* genotype	Δ F508 homozygous
Medical history	Bilateral lung transplantation Pancreatic failure with diabetes
Age at LT (years)	32
Years post-LT (01/12/2014)	3.25
Extubation (days post-LT)	4
Forced expiratory volume (% of the predicted value)	
1 month post-LT	70
3 months post-LT	82
6 months post-LT	80
1 year post-LT	78
2 years post-LT	83
3 years post-LT	90
Time-points of sampling (days post-LT)*	
BA (n=8)	**−1**; **0**; **3**; **5**; **11**; **25**; **95**; 200
BAL (n=3)	25; 200; 718

^*^Samples from which *P. aeruginosa* was isolated are indicated in bold. Samples submitted to 16 S rRNA sequencing and taxonomic analysis are underlined.
