# Supplementary material for: Rapid adaptation drives invasion of airway donor microbiota by Pseudomonas after lung transplantation
Source: Sci Rep. 2017 Jan 17;7:40309. doi: 10.1038/srep40309 (PMC5240337; doi:10.1038/srep40309)
Supplement: Supplementary Data [file srep40309-s1.doc]

**Supplementary information**

**Rapid adaptation drives invasion of airway donor microbiota by *Pseudomonas* after lung transplantation**

M. Beaume, T. Köhler, G. Greub, O. Manuel, JD. Aubert, L. Baerlocher, L. Farinelli, A. Buckling, C. van Delden and the Swiss Transplant Cohort Study

**Supplementary methods**

*Microbiological and clinical data*

Clinical data were collected from one adult CF patient at the Lausanne University Hospital Center (CHUV, Switzerland). Bacterial colonization was determined for conventional diagnostic by the local bacteriology laboratory. All the immunosuppressive treatment periods and rejection events have been recorded in the Swiss Transplant Cohort Study (STCS) database. The treating physician determined the selection and duration of antibiotic treatments. Antibiotic therapy was fully detailed in the local Lausanne University Hospital Center database.

*Total genomic DNA extraction from native samples*

1 mL of airway sample were treated with 100 µg/mL of dithiothreitol (DTT) to dissociate the mucus, and incubated 10 min at 37°C with shaking (220 rpm). 500 µL of these products were 3 times frozen and thawed, and centrifuged for 10 min at 10,000 g (4°C). We combined a chemical and mechanical lysis by adding 180 uL of ATL buffer (DNeasy, QIAGEN) into the 250 µL of the supernatants and running samples into the FastPrep instrument (MP Biomedicals) for 4 x 1 min at 6.5M/S with glass beads (acid-washed G1152, Sigma). After centrifugation (30 s; 10,000 g) to remove beads, samples were submitted to total gDNA extraction by using the DNeasy kit (QIAGEN) according to the manufacturer’s instructions. Isolated gDNA were used for deep sequencing and quantitative real-time PCR (qPCR) studies.

*16S rRNA gene amplification and sequencing*

The V4-V6 regions of the 16S rRNA gene were amplified by PCR using Uni16_518F and Uni16_1064R primers (Table S1). The master mix contained 1X Platinum polymerase buffer, 0.8 units PCR Platinum Taq DNA polymerase high fidelity (Invitrogen), 3.7 mM MgSO4, 200 mM dNTPs (each), and 0.4 uM of each primer. 4 µL of each total gDNA were directly added to reach a final volume of 50 µL. PCR cycling conditions included initial denaturation at 94°C for 3 min; 30 cycles of 94°C for 30 s, 57°C for 45 s, and 72°C for 1 min; and a final extension at 72°C for 2 min. Quality of PCR amplicons was evaluated on 1% agarose gel. Three replicate reactions were performed and pooled before PCR product purification. PCR products were purified by using the GeneJet PCR purification kit (ThermoScientific) following manufacturer’s recommendations and eluted in 40 µL of 0.1X elution buffer.

PCR products were sequenced by using an Illumina MiSeq platform with a 2 x 300-bp protocol (Fasteris SA, Plan-les-Ouates, Switzerland). A custom bioinformatic pipeline performed quality filtering to remove low quality reads. The reads obtained were first mapped on the 18S human rRNA gene by using the BWA software (v0.5.9) to deplete contaminant reads. Then, reads were mapped onto the GreenGenes database ^1^ and assigned to a specific taxon. Diversity and richness index were calculated by using the VEGAN package (http://vegan.r-forge.r-project.org/). Calculation was performed for each sample on 7000 reads taken randomly from the pool of reads.

*Clondiag genotyping*

Genomic DNA of these three isolates was purified by using the DNeasy Blood and Tissue kit (QIAGEN) on pellets of overnight cultures following the recommendations of the manufacturer. gDNA concentrations were estimated by using a Nanodrop ND1000 instrument. 1.85, 0.96 and 1 µg of gDNA of Z(-1)A, Z(-1)B and Z95B isolates respectively were used for the linear amplification and biotin labelling. Hybridization, staining and data acquisition were performed following manufacturer’s recommendations.

*PCR for capillary sequencing or digestions*

All PCR reactions contained dNTPs (0.25 mM final), Taq Buffer (1X final, Sigma), DMSO (5% final concentration), Taq polymerase (2.5U/reaction, Sigma) and primers (0.6 µM each, Table S1). 2.4 µL of bacterial lysate served as template in a final volume of 30 µL. PCR conditions were as described in Table S2. PCR products were purified by using GeneJet PCR purification kit (ThermoScientific) and send to sequencing (Fasteris SA, Plan-les-Ouates, Switzerland) or digested directly as described in Table S2. All reactions were performed in a final volume of 25 µL following the manufacturer’s recommendations. Products of digestion were visualized on 2% agarose gel and compared with a non-digested control of each reaction.

*Genome sequencing of clinical isolates*

Genomic DNA were sequenced on an Illumina HiSeq 2500 platform generating 2 x 100-bp paired-end reads (Fasteris SA, Plan-les-Ouates, Switzerland). We used a multiplex protocol allowing to sequence eight genomic libraries on one line of the flow cell.

Sequence reads from the Z95B isolate were *de novo* assembled using Velvet ^2^ (version 1.2.07). The pair-read insert average size was set at 300 bases and the mate-pair insert average size was set at 5K bases, both with a standard deviation of 10%. A BWA (v0.5.9) ^3^ mapping using 1M reads was carried on the *de novo* contigs to validate the *de novo* assembly. Reads obtained from Z(-1)A and Z(-1)B gDNA sequencing were mapped onto the *de novo* assembled genome of the Z95B isolate, and SAMTOOLS was used for the variant calling (<http://samtools.sourceforge.net/>) (Table S5).

*Mutation analysis by deep sequencing*

PCR reaction contained dNTPs (0.2 mM final), Taq Buffer (1X final, Sigma), DMSO (5% final concentration), Taq polymerase (2.5U/reaction, Sigma) and primers (0.6 µM each). Specific primers were distributed as follows: multiplex 1: *phoP*, *ftsY*, PA4435; multiplex 2: *gidA*, *azu*; multiplex 3: PA1550, PADK2-14410, *hxcS*; multiplex 4: PA2061, *trpF* promoter region, PA4949; multiplex 5: PA4288, *phoQ*, *mutL* (Table S1). 4 µL of total gDNA served as template in a final volume of 50 µL. PCR cycling conditions included initial denaturation at 95°C for 2 min; 30 cycles of 95°C for 20 s, 57°C for 30 s, and 72°C for 30 s; and a final extension at 72°C for 4 min. PCR products were purified by using the GeneJet PCR purification kit (ThermoScientific).

Purified PCR products were pooled per time-point (date of sampling) and sequenced on an Illumina HiSeq 2500 platform with 1 x 100 cycles (Fasteris SA, Plan-les-Ouates, Switzerland). The six libraries were sequenced in one FlowCell lane. Reads obtained were mapped onto the reference sequence of PAO1 genome by using the BWA tool (v 0.5.9) and SNP and Indel calling was performed by using SAMTOOLS v 0.1.18 and BCFtools (<http://samtools.sourceforge.net/>). The variant caller of SAMTOOLS relies on a Bayesian model to measure the quality of a called consensus. Reads mapping quality was capped at 60 in the calling process to avoid bias toward high values. The filtering of SNP and InDel (Insertion/Deletion) was performed with the following criteria: i) Coverage threshold of 10, ii) SNP or InDel supported by at least 3 reads in forward and 3 reads in reverse, iii) Variant quality greater than 25. Additional criteria were used for SNP only: i) Minimum root mean square (RMS) of the mapping qualities of the reads overlapping the position of 25, ii) No InDel within 10 bp, iii) Maximum 2 SNPs within 10 bp window. Additional criteria were used for InDel only: i) Minimum RMS of the mapping qualities of the reads overlapping the position of 10, ii) No InDel within 30 bp.

The number of reads mutated at the position previously identified in the Z95B genome was count. Results are expressed as a percentage of mutated reads in the total read population.

*Microtiter dish biofilm assay*

Overnight cultures were grown in Lysogeny Broth (LB) medium at 37°C with shaking. After incubation, overnight cultures were centrifuged 3 min at 6000×g, and pellets were resuspended in 0.9% NaCl to discard alginate residues. Bacterial suspensions were then diluted to OD_600_=2 and 100 µL were used to inoculate 900 µL of M63 medium supplemented with 0.2% glucose, 2mM MgSO_4_ and 0.05% Casamino acids in a 24-well polystyrene microtiter dish (Costar). Two duplicates were performed. Microtiter dishes were incubated at 30°C without shaking for 24h. Cultures were stained with 0.1% crystal violet, followed by two washes with water. The dye was solubilised in 40% ethanol and absorbance measured at 590 nm in a plate reader (BioTek Synergy H1).

**Supplementary tables**

**Supplementary Table S1. Relative abundance of the *Mycobacterium* genus in airway samples**

| **Days post-LT** | | **-1** | **0** | **11** | **25** | **95** | **200** | **718** |
| --- | --- | --- | --- | --- | --- | --- | --- | --- |
| *% of Mycobacterium* | **BA** | 1.22 | 1.34 | 0.47 | 0.22 | 1.32 | 0 |  |
|  | **BAL** |  |  |  | 0.02 |  | 0.08 | 0 |

BA: bronchial aspirate, BAL: bronchoalveolar lavage

**Supplementary Table S2**. Genome assembly and read mapping data.

| Genome | Z(-1)A | Z(-1)B | Z95B |
| --- | --- | --- | --- |
| Assembly | | | |
| Number of contigs |  |  | 64 |
| Sum of contig length |  |  | 6'733'668 |
| Mapping | | | |
| Number of reads | 9'319'242 | 5'670'274 | 6'365'050 |
| Number of mapped reads | 9'180'092 | 5'574'469 | 6'303'541 |
| % of mapped reads | 98.51 | 98.31 | 99.03 |

**Supplementary Table S3. List of SNPs and indels found by comparison between Z(-1)A, Z(-1)B and Z95**B genomes.

| **Mutated genome** | | | **Reference genome** | | | | | | **Type of mutation** | **Synonymous / Non-synonymous** |
| --- | --- | --- | --- | --- | --- | --- | --- | --- | --- | --- |
| **Name** | **Mutation**  **(nucleotide)** | **Mutation**  **(protein)** | **Name^†^** | **Position** | **Nucleotide sequence** | **Protein sequence** | **Gene name** | **Product** |
| **Z(-1)A** | TAC | Y | PAO1 | 4144293 | TCC | S | *wspR* | diguanylate cyclase activity | SNP | NS |
| **Z(-1)A** | ACA | T | PAO1 | 3948720 | GCA | A | *rnt* | ribonuclease T | SNP | NS |
| **Z(-1)A** | GTC | V | PAO1 | 5828995 | GCC | A | PA5176 | HP | SNP | NS |
| **Z(-1)A** | GTG | V | PAO1 | 370110 | GCG | A | *aaaA* | arginine-specific autotransporter | SNP | NS |
| **Z(-1)A** | CTG | L | PAO1 | 5550546 | CCG | P | *mutL* | DNA mismatch repair protein MutL | SNP | NS |
| **Z(-1)A** | AAC | N | PAO1 | 5334265 | GAC | D | *glmM* | phosphoglucosamine mutase | SNP | NS |
| **Z(-1)A** | ATC | I | PAO1 | 991044 | ACC | T | *rsmA* | carbon storage regulator | SNP | NS |
| **Z(-1)A** | AAG | K | PAO1 | 1024193 | GAG | E | *relA* | GTP pyrophosphokinase | SNP | NS |
| **Z(-1)A** | AGC | S | PAO1 | 1056841 | GGC | G | *tolB* | transporter activity | SNP | NS |
| **Z(-1)A** | CTG | L | PAO1 | 14520546 | CCG | P | PA1399 | probable transcriptional regulator | SNP | NS |
| **Z(-1)A** | AGC | S | PAO1 | 4954157 | GGC | G | *ftsI* | penicillin-binding protein 3 | SNP | NS |
| **Z(-1)A** | CAC | H | PAO1 | 2518784 | CGC | R | *gcd* | glucose dehydrogenase | SNP | NS |
| **Z(-1)A** | TTT | F | PAO1 | 704926 | TTG | L | *trpD* | anthranilate phosphoribosyltransferase | SNP | NS |
| **Z(-1)A** | ATT | I | PAO1 | 4595146 | AAT | N | *ampC* | beta-lactamase precursor | SNP | NS |
| **Z(-1)A** | TAC | Y | PAO1 | 4594671 | CAC | H | *ampC* | beta-lactamase precursor | SNP | NS |
| **Z(-1)A** | ACG | T | PAO1 | 5560304 | GCG | A | PA4955 | HP | SNP | NS |
| **Z(-1)A** | GGT | G | PAO1 | 1114058 | GGC | G | PA1027 | probable aldehyde dehydrogenase | SNP | S |
| **Z(-1)A** | ATT | I | PAO1 | 1690373 | ATC | I | *coop1 / coop2* | cytochrome c oxidase | SNP | S |
| **Z(-1)A** | GGA | G | PAO1 | 4629057 | GGC | G | *tyrS* | tyrosyl-tRNA synthetase | SNP | S |
| **Z(-1)A** | CTT | L | PAO1 | 1299779 | CTC | L | PA1198 | cell wall-associated hydrolase | SNP | S |
| **Z(-1)A** | TCA | S | PAO1 | 2332656 | TCG | S | PA2120 | HP | SNP | S |
| **Z(-1)A** | A |  | PAO1 | 2163879 | G |  | Intergenic |  | SNP |  |
| **Z(-1)A** | T |  | PAO1 | 4295955 | C |  | Intergenic |  | SNP |  |
| **Z(-1)A** | T |  | PAO1 | 1117952 | C |  | Intergenic |  | SNP |  |
| **Z(-1)A** | A |  | PAO1 | 5289153 | G |  | Intergenic |  | SNP |  |
| **Z(-1)A** | A*G | L* + frameshift | PAO1 | 4482028-4482018 | ATGCGATCTCGCG | LDAISRP | *sltB1* | soluble lytic transglycosylase B | Deletion | NS |
| **Z(-1)A** | C*C | Y* + frameshift | PAO1 | 4265515-4265511 | CGAGCGC | YERP | PA3806 | HP | Deletion | NS |
| **Z(-1)A** | GCG*CAT | VR + frameshift | PAO1 | 4006965-4006968 | GCGGCTGCAT | VRLH | *nalD* | transcriptional regulator | Deletion | NS |
| **Z(-1)A** | AGC*GCGAG | SA + frameshift | PAO1 | 3480891 | AGCGGCGAG | SGE | *xcpS* | general secretion pathway protein F | Deletion | NS |
| **Z(-1)A** | GC*GGACAAG | ERT + frameshift | PAO1 | 3338469-3338466 | GCGGCTGGACAAG | ERLDK | PA2980 | HP | Deletion | NS |
| **Z(-1)A** | TGGG*GCGCC | WG + frameshift | PAO1 | 5818227 | TGGGCGCGCC | WARL | *dctQ* | dicarboxylic acid transport | Deletion | NS |
| **Z(-1)A** | TC*GC | W* + frameshift | PAO1 | 5065310-5065295 | TCCGTGGCGTCCGCCACTGC | WVRGVRHCP | *ampD* | beta-lactamase expression regulator AmpD | Deletion | NS |
| **Z(-1)A** | GCC*ACC | A*T | PAO1 | 452678-452683 | GCCGACCAGACC | ADQT | *pilJ* | twitching motility protein PilJ | Deletion | NS |
| **Z(-1)A** | TTCAT*GCAAC | F* + frameshift | PAO1 | 2143895-2143896 | TTCATCGGCAAC | FIGN | *bacA* | bacitracin resistance protein | Deletion | NS |
| **Z(-1)A** | * | Del + frameshift after 17 a.a. | PAO1 | 4145747-4145893 | 146 nts |  | *wspF* | probable methylesterase | Deletion | NS |
| **Z(-1)A** | * |  | PAO1 | 2728308-2733340 | 5032 nts |  | *bexR*, PA2433, PA2434, PA2435, PA2436, PA2437 |  | Deletion | NS |
| **Mutated genome** | | | **Reference genome** | | | | | | **Type of mutation** | **Synonymous / Non-synonymous** |
| **Name** | **Mutation**  **(nucleotide)** | **Mutation**  **(protein)** | **Name^1^** | **Position** | **Nucleotide sequence** | **Protein sequence** | **Gene name** | **Product** |
| **Z(-1)A** | C*A |  | PAO1 | 831168-831185 | CGCACGTCACGAGCGAGGAA |  | Intergenic, promoter region of *algU* |  | Deletion |  |
| **Z(-1)A** | CCGCCCCGCC | VAPP + frameshift | PAO1 | 4265896 | CCG*CC | VAAM | PA3806 | HP | Insertion | NS |
| **Z(-1)A** | TGGAGTGCTG | VEC + frameshift | PAO1 | 5680952 | TG*CTG | VLL | *ponA* | penicillin-binding protein 1A | Insertion | NS |
| **Z(-1)A** | AGCATCTAATCGC | SI stop | PAO1 | 1911947 | AGCATC*AATCGC | SINR | PA1767 | HP | Insertion | NS |
| **Z(-1)A** | CTGGCTGGCCGG | SWLAG + frameshift | PAO1 | 562187 | CT*GG | S*W | PA0502 | probable biotin biosynthesis protein bioH | Insertion | NS |
| **Z(-1B)** | CCC | P | PAO1 | 1454081 | ACC | T | PA1339 | amino acid ABC transporter ATP binding protein | SNP | NS |
| **Z(-1B)** | AGC | S | PAO1 | 4594770 | GGC | G | *ampC* | beta-lactamase precursor | SNP | NS |
| **Z(-1B)** | GGG | G | PAO1 | 3891049 | GTG | V | *rhlB* | rhamnosyltransferase chain B | SNP | NS |
| **Z(-1B)** | CGG | R | PAO1 | 670757 | CGC | R | PA0608 | probable phosphoglycolate phosphatase | SNP | S |
| **Z(-1B)** | CAA | Q | PAO1 | 819374 | CAG | Q | PA0751 | putative ammonia monooxygenase | SNP | S |
| **Z(-1B)** | T |  | PAO1 | 6125632 | A |  | Intergenic |  | SNP |  |
| **Z(-1B)** | A |  | PAO1 | 5065348 | G |  | Intergenic |  | SNP |  |
| **Z(-1B)** | ACC*GGT | T* + frameshift | PAO1 | 1730625-1730635 | ACCATCGACCCGCTGGT | TIDPLV | *sucC* | succinyl-CoA synthetase beta chain | Deletion | NS |
| **Z(-1B)** | CTGC*AACTG | L*QL | PAO1 | 4993531-4993533 | CTGCTGCAACTG | LLQL | *rpoN* | RNA polymerase sigma-54 factor | Deletion | NS |
| **Z(-1B)** | AAGCGCGC | KRARE + frameshift | PAO1 | 475137 | AA*GC | KRAQ | *mexB* | Resistance-Nodulation-Cell Division (RND) multidrug efflux transporter MexB | Insertion | NS |
| **Z(-1B)**  **Z95B** | GAT | D | PAO1 | 991056 | GGT | G | *rsmA* | carbon storage regulator | SNP | NS |
| **Z(-1B)**  **Z95B** | CAT | H | PAO1 | 628985 | CAC | H | PA0574 | HP | SNP | S |
| **Z(-1B)**  **Z95B** | A |  | PAO | 5289158 | G |  | Intergenic |  | SNP |  |
| **Z(-1B)**  **Z95B** | C |  | PAO1 | 99784 | G |  | Intergenic |  | SNP |  |
| **Z(-1B)**  **Z95B** | GTC*ACAAG | VT + frameshift | PAO1 | 3525268 | GTCAACAAG | VNK | *wbpM* | nucleotide sugar epimerase/dehydratase WbpM | Deletion | NS |
| **Z(-1B)**  **Z95B** | GTGC*GACCTA | V*R + frameshift | LESB58 | 4602158-4602154 | GTGCTACTGGACCTA | VLLDL | *pys2* | pyocin S2 | Deletion | NS |
| **Z(-1B)**  **Z95B** | TTCA*GCGGA | FK*G | PAO1 | 4988842-4988840 | TTCAAGCGCGGA | FKRG | PA4456 | probable ATP-binding component of ABC transporter | Deletion | NS |
| **Z(-1B)**  **Z95B** | AGGGGG | P*P + frameshift | PAO1 | 1544522-1544540 | AGGCCAGGCCGGCTGCCGCGCTGGG | PSAAAGLA | PA1419 | probable transporter | Deletion | NS |
| **Z95B** | TGC | C | PAO1 | 6258921 | CGC | R | *gidA* | glucose-inhibited division protein A | SNP | NS |
| **Z95B** | CTG | L | PAO1 | 5554920 | CCG | P | PA4949 | predicted sugar kinase | SNP | NS |
| **Z95B** | GTC | V | PAO1 | 4968082 | GCC | A | PA4435 | probable acyl-CoA dehydrogenase | SNP | NS |
| **Z95B** | ACC | A | PAO1 | 4810781 | GCC | T | PA4288 | probable transcriptional regulator | SNP | NS |
| **Z95B** | CCC | P | PAO1 | 746649 | CTC | L | *hxcS* | probable type II secretion system protein | SNP | NS |
| **Z95B** | GGC | G | PAO1 | 1277877 | CGC | R | *phoP* | two-component response regulator PhoP | SNP | NS |
| **Z95B** | CAC | H | PAO1 | 1687578 | CGC | R | PA1550 | HP | SNP | NS |
| **Z95B** | AAT | N | PAO1 | 2258420 | AAC | N | PA2061 | probable ATP-binding component of ABC transporter | SNP | S |
| **Z95B** | GAT | D | PAO1 | 5521772 | GAC | D | *azu* | azurin precursor | SNP | S |
| **Z95B** | T |  | PAO1 | 1797851 | G |  | Intergenic |  | SNP |  |
| **Z95B** | A |  | PAO1 | 3328964 | G |  | Intergenic |  | SNP |  |
|  |  |  |  |  |  |  |  |  |  |  |
| **Mutated genome** | | | **Reference genome** | | | | | | **Type of mutation** | **Synonymous / Non-synonymous** |
| **Name** | **Mutation**  **(nucleotide)** | **Mutation**  **(protein)** | **Name^1^** | **Position** | **Nucleotide sequence** | **Protein sequence** | **Gene name** | **Product** |
| **Z95B** | C*C |  | PAO1 | 3493825-3493823 | CCCGC |  | Intergenic |  | Deletion |  |
| **Z95B** | G*C | A* + frameshift | LESB58 | 411614 | GCCGAGCC | AAEP | *ftsY* | signal recognition particle receptor FtsY | Deletion | NS |
| **Z95B** | A*C | L*V | DK2 | 3113153 | AACGGCAGCTTGAC | LNGSLTV | DK2_14410 | arac family transcriptional regulator | Deletion | NS |
| **Z95B** | TGTGTGG | YL* + frameshift | PAO1 | 1278863 | TGTG*G | YLWL | *phoQ* | two-component sensor PhoQ | Insertion | NS |

HP, hypothetical protein

* indicates the position of the mutation

† RefSeq accession number: NC_002516 (PAO1), NC_018080 (DK2), NC_011770 (LESB58)

a.a.: amino acid

**Supplementary Table S4.** Minimal inhibitory concentrations

|  | **MIC (µg/mL)** | | | | |
| --- | --- | --- | --- | --- | --- |
|  | **Azithromycin** | **Imipenem** | **Amikacin** | **Tobramycin** | **Colistin** |
| Z(-1)A | 1024 / 1024 | 4 / 8 | 8 / 4 | 0.5 / 0.5 | 0.5 / 0.5 |
| Z(-1)B | 4096 / 2048 | 32 / 32 | 32 / 16 | 1 / 1 | 1 / 0.5 |
| Z0A | 1024 / 1024 | 4 / 8 | 8 / 4 | 0.5 / 0.5 | 0.5 / <0.25 |
| Z3B | 4096 / 512 | 32 / 32 | 32 / 32 | 1 / 1 | 4 / 0.5 |
| Z5A | 1024 / 1024 | 16 / 16 | 32 / 16 | 1 / 1 | 0.5 / <0.25 |
| Z5B | 4096 / 4096 | 32 / 32 | 16 / 32 | 1 / 1 | 1 / 0.5 |
| Z11A | 512 / 512 | 16 / 16 | 32 / 16 | 1 / 1 | 1 / 2 |
| Z11B | 4096 / 4096 | 32 /32 | 16 / 32 | 1 / 1 | 1 / 0.5 |
| Z25B | 4096 / 2048 | 32 / 32 | 32 / 32 | 1 / 1 | 1 / 0.5 |
| Z95B | 4096 / 4096 | 32 / 32 | 64 / 32 | 2 / 4 | >16 / >16 |
| Reference strain PAO1 | 128 / 128 | 1 / 1 | 2 / 1 | 0.25 / 0.25 | 1 / 1 |
| EUCAST breakpoint (v_5.0) | - | S≤4, R>8 | S≤8, R>16 | S≤4, R>4 | S≤4, R>4 |

Values obtained with the two biological replicates are separated by a slash.

**Supplementary Table S5. Primer sequences**

| **Primer** | **Targeted gene** | **Sequence (5’ - 3′)** | **Reference** |
| --- | --- | --- | --- |
| Uni16_518F | 16S rRNA | CCAGCAGCYGCGGTAAN | 4 |
| Uni16_1064R | 16S rRNA | CGACRRCCATGCANCACCT | 4 |
| rpsL-F | *rpsL* | GCAAGCGCATGGTCGACAAGA | 5 |
| rpsL-R | *rpsL* | CGCTGTGCTCTTGCAGGTTGTGA | 5 |
| phoP_121_F | *phoP* | AGCGAATACCACCACGACC | This work |
| phoP_240_R | *phoP* | GGCGGTGAGGATCAGGATC | This work |
| phoQ_464_F | *phoQ* | GTCAGCGAGTTCGAGAGCCT | This work |
| phoQ_558_R | *phoQ* | TCAGACCCAGCCAGAGCAG | This work |
| gidA_223_F | *gidA* | GACAAGGGCGGTATCCAGTT | This work |
| gidA_325_R | *gidA* | GGATAGCGGCCTTATAGAGCA | This work |
| PA4288_198_F | PA4288 | GGTATGGATACCACCGGAGC | This work |
| PA4288_295_R | PA4288 | GCAGGCGTCGTGACAGTTC | This work |
| Azu_259_F | *azu* | GGCCTGGACAAGGATTACCT | This work |
| Azu_387_R | *azu* | CATGTACTGCTCGCCTTCCTT | This work |
| PA1550_295_F | PA1550 | CTCAACCTGATCTCGCCGA | This work |
| PA1550_389_R | PA1550 | GTCAGTTGCCCGACGTAGC | This work |
| PA2061_654_F | PA2061 | CGATCGGGTCTGCGTGAT | This work |
| PA2061_752_R | PA2061 | ATCAGCAGGCGCGTGTAG | This work |
| i_trpF_-87_F | *trpF* promoter region | CGGCTATCGCGAAATATCTG | This work |
| i_trpF_R | *trpF* promoter region | ACGAGACATGATGTGACGACG | This work |
| PA4949_97_F | PA4949 | CCGGGCTTCGAACTCATG | This work |
| PA4949_228_R | PA4949 | CGCGATCAGGTAGCCGTC | This work |
| PADK2_14410_87_F | PADK2-14410 | AGATTGGCATAGCCACATGG | This work |
| PADK2_14410_189_R | PADK2-14410 | GATGCAGATGGCCCTTGC | This work |
| FtsY_285_F | *ftsY* | GTCGGCCGAGGAGCATTC | This work |
| FtsY_429_R | *ftsY* | CTCGCTGACTGCCGGAGG | This work |
| mutL_1041_F | *mutL* | GACCAGCCTGACCGAGCC | This work |
| mutL_1169_R | *mutL* | GATGAGCCGCCACTCCAG | This work |
| PA0687_822_F | *hxcS* | CTCCACCCTGGCGATCCT | This work |
| PA0687_956_R | *hxcS* | GAGACACCTTCGCGAACCTG | This work |
| PA1419_901_F | PA1419 | CTGCCGGTATGCCTGATCG | This work |
| PA1419_1345_R | PA1419 | CCAGTTCGCCGAGCGGAC | This work |
| sucC_106_F | *sucC* | GACAAGATCGGCGGCAGC | This work |
| sucC_564_R | *sucC* | GAACAGCTTCGCCAGGCC | This work |
| dctQ_-57_F | *dctQ* | GACCTGATCAAGGCCGCC | This work |
| dctQ_389_R | *dctQ* | CAGAGCAGGCCGGCGTAG | This work |
| wspF-1 | *wspF* | TGGTGTCCTACAAGGATCGTGA | This work |
| wspF-2 | *wspF* | TCATGAGGGTTGTGCATGTTTC | This work |
| 208 |  | ACGGCCGACC | 6 |
| PA4435_337_F | PA4435 | AAGTACCTGCCGAAGCTGGT | This work |
| PA4435_428_R | PA4435 | GTCTTCACCCCTTGCAGGTC | This work |
| Las_F1 | *lasR* | gtgggctgactggacatctt | This work |
| LasR8 | *lasR* | ctgcggcagtcgtttcgagaat | This work |

**Supplementary Table S6**. PCR and digestion conditions.

| **PCR** | | | | | | | | |
| --- | --- | --- | --- | --- | --- | --- | --- | --- |
| Genes targeted | *gidA, mutL, phoP, ftsY,* DK2-14410*,* PA4949*,* PA4435*,* PA4288*, hxcS, sucC*, PA1419 | | *phoQ*, PA1550 | | *dctQ* | | *wspF, lasR* | |
| PCR cycling conditions | 95°C 2min |  | 95°C 2min |  | 95°C 2min |  | 95°C 2min |  |
|  | 95°C 20s  57°C 30s  72°C 30s | 27 cycles | 95°C 20s  60°C 30s  72°C 30s | 40 cycles | 95°C 20s  57°C 30s  72°C 3min | 27 cycles | 95°C 20s  57°C 30s  72°C 1min 30s | 27 cycles |
|  | 72°C 4min |  | 72°C 4min |  | 72°C 4min |  | 72°C 4min |  |
| **Digestion** | | | | |  | | | |
| Genes targeted | *sucC* | *dctQ* | PA1419 | *wspF* |  |  |  |  |
| Restriction enzyme (NEB) | XcmI | NarI | NaeI | BstEII |  |  |  |  |
| Volume of PCR product digested (µL) | 8 | 4 | 5 | 5 |  |  |  |  |
| Incubation | 37°C 1h | 37°C 1h30 | 37°C 2h | 60°C 2h |  |  |  |  |

**Supplementary Table S7. Sequence files**

|  | **Reference** | **Corresponding sample** |
| --- | --- | --- |
| **Submission PRJEB14611: Deep sequencing on the *P. aeruginosa* population** | HAK-111 and HAK-122 | BA; day -1 |
|  | HAK-123 | BA; day 0 |
|  | HAK-124 | BA; day 11 |
|  | HAK-125 | BA; day 25 |
|  | HAK-126 and HAK-112 | BA; day 95 |
| **Submission PRJEB13210: 16S rRNA gene sequencing** | HAK-15 | BA; day -1 |
|  | HAK-16 | BA; day 0 |
|  | HAK-128 | BA; day 3 |
|  | HAK-17 | BA; day 11 |
|  | HAK-18 | BA; day 25 |
|  | HAK-20 | BA; day 95 |
|  | HAK-21 | BA; day 200 |
|  | HAK-19 | BAL; day 25 |
|  | HAK-22 | BAL; day 200 |
|  | HAK-23 | BAL; day 718 |
| **Submission PRJEB14598: whole genome sequencing** | HAK-5 | Z(-1)A |
|  | HAK-6 | Z(-1)B |
|  | HAK-7 and HAK-10 | Z95B |

**Supplementary Figure**


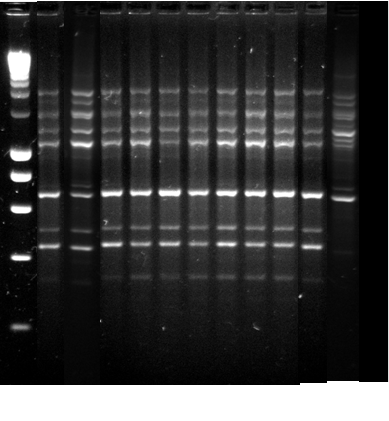


200

400

600

800

1500

2000

1000

Z(-1)A

Ladder

Z(-1)B

Z0A

Z3B

Z5A

Z5B

Z11A

Z11B

Z25B

Z93B

PA14

-

| Clondiag | 2C22 | 2C22 | nd | nd | nd | nd | nd | nd | nd | 2C22 |  |  |
| --- | --- | --- | --- | --- | --- | --- | --- | --- | --- | --- | --- | --- |
| lasR sequence | N214D | N214D | nd | N214D | nd | N214D | nd | N214D | nd | N214D |  |  |

**Supplementary Figure S1. RAPD fingerprints of *P. aeruginosa* isolates.**

Red arrows highlight 8 particular bands shared by all Z isolates indicating that they have the same genotype. This 2% agarose-gel was stained with SybrSafe DNA gel stain (Invitrogen). Molecular size marker was run in lane “Ladder” and its size (in kilobases) are indicated to the right of the lane (Smart Ladder, Eurogentec). PA14 isolate was used as reference strain. The symbol “-“ corresponds to the negative control of the amplification reaction. nd= not determined.

Reference List

1 DeSantis, T. Z. *et al.* Greengenes, a chimera-checked 16S rRNA gene database and workbench compatible with ARB. *Applied and environmental microbiology* **72**, 5069-5072, doi:10.1128/AEM.03006-05 (2006).

2 Zerbino, D. R. & Birney, E. Velvet: algorithms for *de novo* short read assembly using de Bruijn graphs. *Genome research* **18**, 821-829, doi:10.1101/gr.074492.107 (2008).

3 Li, H. & Durbin, R. Fast and accurate long-read alignment with Burrows-Wheeler transform. *Bioinformatics* **26**, 589-595, doi:10.1093/bioinformatics/btp698 (2010).

4 Filkins, L. M. *et al.* Prevalence of streptococci and increased polymicrobial diversity associated with cystic fibrosis patient stability. *J.Bacteriol.* **194**, 4709-4717 (2012).

5 Dumas, J. L., van Delden, C., Perron, K. & Kohler, T. Analysis of antibiotic resistance gene expression in *Pseudomonas aeruginosa* by quantitative real-time-PCR. *FEMS microbiology letters* **254**, 217-225, doi:10.1111/j.1574-6968.2005.00008.x (2006).

6 Mahenthiralingam, E., Campbell, M. E., Foster, J., Lam, J. S. & Speert, D. P. Random amplified polymorphic DNA typing of *Pseudomonas aeruginosa* isolates recovered from patients with cystic fibrosis. *Journal of clinical microbiology* **34**, 1129-1135 (1996).
